# A retrospective study on side effects of first-line antiretroviral drugs on HIV patients based on 1A, 2A, and 5A regimen records at Zomba Central Hospital, Malawi

**DOI:** 10.4314/ahs.v23i3.54

**Published:** 2023-09

**Authors:** Ruth Sindie, Elias Mwakilama, Pachalo Chizala, Jimmy Namangale

**Affiliations:** 1 SouthWest University, China; 2 University of Malawi, Zomba, Malawi; 3 National Statistical Office, Zomba, Malawi

**Keywords:** HIV/AIDS, ARV regimens, symptoms, side effects, socio-demographics, multinomial logistic regression

## Abstract

AIDS is an incurable disease that is common in Africa. Patients with HIV/AIDS having a CD4 count of less than 240 are put on life prolonging ARV drugs. The ARVs have serious side effects on some patients which may be handled by treating them or switching patient's drug to one with no or less serious side effects. However, before doing this, more understanding of the circumstances that lead to a side effect is vital. We use statistical analyses to link side effects of 1A, 2A, and 5A treatment regimens to the patient's social and demographic characteristics based on hospital data records. A retrospective review of patients' master cards (2011-2014) was done to assess adverse effects associated with different ARV regimens. Out of the 901 patients that showed side effects, 65.37% were females aged 31-40 and 34.63% were males. Comparatively, 1A regimen showed more side effects than 2A and 5A regimens. Age, gender and occupation correlated significantly with regimen symptoms (p< 0.05). Unlike men, women had the following extra side effects; cough, peripheral neuropathy and leg pains as compared to lipodystrophy. Our results show that old people (50years+) are less likely to get skin rash and other symptoms compared to lipodystrophy (RRR=0.973). Further, the probability of either having cough (0.0021, p< 0.05), or skin rash (0.0021, p< 0.05), as a side effect, on average, decreases as age increases with the same sex and weight. The probability of having peripheral neuropathy (0.0042, p< 0.01), however, increases with age. Knowledge of HIV patient's socio-demographics and the patient's regimen side effects can be utilised to appropriately manage severe ARV side effects. A therapy consideration that takes into account chemicals in ARV regimen responsible for specific side effects can be directed to patients with compatible socio-demographic characteristics.

## Background

Acquired immunodeficiency syndrome (AIDS) was first observed in 1959 in Congo, Africa, and, later in 1981, in the United States of America (USA). In the 1980s, AIDS was initially observed among homosexual men who were reported with cases of Kaposi's sarcoma and pneumocystis carinii (Avert, 2017). As such, people had the initial perception of AIDS as a “gay” disease, and this delayed testing and drug development. This also led to the spread of the disease to heterosexuals and haemophiliacs through blood transfusion (Avert, 2017).

In 1983, the Human Immunodeficiency Virus (HIV) was discovered (Geubbels & Bowie, 2006). Since then, HIV and AIDS have become a major epidemic. Globally, it is estimated that over 37.9 million people are living with HIV with 40% of the new infections being among the people below 25 years of age (Avert, 2018). From the 37.9 million people, about 68% are from the Sub-Saharan Africa, approximately 25.8 million people. More than half of the new infections occur among the youths aged 15-24 years with teenage girls being 5 to 6 times more likely to be infected compared to their male counterparts (KFF, 2014). According to Williams (2014), the epidemic of HIV in Malawi started earlier than in most southern Africa. To date, Malawi has around 1.1 million people living with HIV and AIDS (Avert, 2018) with a first case that was first documented in 1982 (Geubbels & Bowie, 2006) and confirmed in May 1985 (Mwale, 2002).

The high mutation rate of HIV hindered the development of an effective vaccine and the first antiretroviral drugs (ARVs) became available in 1995 (Avert, 2017). Currently, over 50 percent of those infected by HIV now have access to treatment globally (Avert, 2018). The number of those infected having access to treatment has also increased in Africa from 53 percent in 2010 to 68 percent in 2012. In Malawi, the population that has access to ARVs is approximated to be increasing annually by 13 percent (Avert, 2018).

However, just like most medicines, ARVs can cause side effects even when taken as PEP 1 (Cai, Xiao, & Zhang, 2014). These unwanted side effects are often mild but can at times be more serious and be of a major impact on the health and quality of life of people. Once started, ARVs must be taken daily for life because every missed dose increases the risk of the drug stopping working (Avert, 2012). For this reason, it is important that people receiving the treatment get all the right help in order to minimize the impact of these side effects.

Different countries have different kinds of ARVs offered to patients. In Malawi, the drugs offered are categorised as First Line and Second Line (Ministry of Health, 2008a). Patients with a cluster of differentiation 4 (CD4) count of less than 250 are initiated on the first line drugs. It is, therefore, a must that a patient starts with the drugs that are in the first line. If patients confirm treatment failure on the first line drugs, then they are switched to the second line drugs.

There are ten different drug combinations called regimens that make up the first- and second-line drugs. These regimens are coded 0 to 9 where combinations coded 0 to 6 belong to the first line and combinations 7 to 9 belong to the second line. Each of these regimens has an adult and paediatric formulation. For instance, a combination of stavudine, lamivudine and nevirapine, is coded 1 with the letter A as 1A to signify an adult formulation and coded 1with a P as 1P for a paediatric formulation (Ministry of Health, 2008b; Montessori, Press, Harris, Akagi, & Montaner, 2004).

When ARV drugs were first introduced in Malawi, almost all adult patients that were starting ARVs were initiated on 1A, a combination of stavudine, lamivudine and nevirapine. Thus, 1A was considered as a default regimen for everyone. Once patients showed some unbearable side effects, they were moved to any of the remaining five first line regimens. In 2013, there was a switch of the default regimen from a combination of stavudine, lamivudine and nevirapine (1A regimen) to a combination of tenofovir, efavirenz and lamivudine (5A regimen) and a combination of zidovudine, lamivudine and nevirapine (2A regimen). This was done after research showed that adults, unlike children on ARV reacted to stavudine which was found in the 1A and 3A regimens. Thus, both 1A and 3A regimens were phased out in adult patients.

Regardless of all the changes, there are still side effects observed in patients. In 2014, the long-hidden side effects of 5A started appearing with some extreme cases of men developing women-like breasts (Nkawihe, 2014). In addition to that, other common side effects that were seen in patients taking 1A were also seen in patients that are on 5A and 2A (Nkawihe, 2014).

This study addresses specifically the following important questions: (1) Do the side effects of 5A regimen significantly differ from those of 1A and 2A? (2) Could the cases of extreme side effects in some HIV patients be due to individual patient characteristics? (3) If so, what are the key socio-demographic factors matching the varying experience of side effects?

The rest of the paper is organised as follows. In section 2, we present the study design, the setting, and the data handling techniques as well as other general approach to the study. In section 3, the findings of the study are discussed while being mindful of the study limitations that come in section 4. Finally, in section 5, we devote our attention to the conclusions and recommendations based on how regimens 1A, 2A, and 5A side effects differ, and their apparent pointer to various socio-demographic factors of patients.

## Methods

### Study Design and Setting

**Figure 1:**
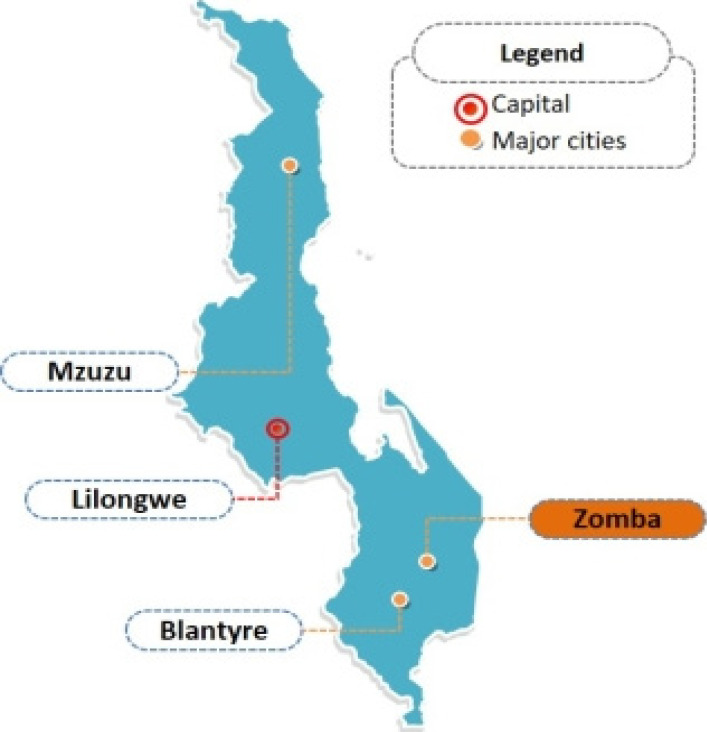
Map-of-Malawi-showing-the-location-of-Zomba-District-in-Southern-Malawi

This was a cross-sectional study conducted by a retrospective review of four years' (2011-2014) patient medical records at the Antiretroviral Therapy (ART) clinic of Zomba Central Hospital (ZCH). The hospital has a catchment area of five traditional authorities (T/As) Chikowi, Malemia, Kuntumanji, Mlumbe, and Mwambo, accounting for human population of at least 851,737 (NSO, 2019). Zomba district is located in the south-eastern part of Malawi (Figure 1). Zomba is included among the districts reported to have higher HIV prevalence rates (13.5%) in Malawi than other districts (Nutor et al., 2020).

This study focused on the patients that were initiated to 1A, 2A and 5A regimens. As such, we only considered data of both youth and adult patients (≥ 14), weighing 25 Kgs and above, initiated on the ART in the period between 2011 and 2013, who had at least one follow up clinical visit after commencing ARVs between 2011 and December 2014. The ART master cards [see Appendix] were used for extracting patients' socio-demographic characteristics, any adverse side effects, regimen switch or interruption of treatment.

### Ethical Considerations

Ethical approval to use data from an ART clinic was sought and given by the Zomba Central Hospital (ZCH) directorate and that it was to be used for purely academic purposes. Cognisant of the fact that the information on patients, especially those on ART is confidential, only patients' masked identities were captured. This was to ensure that the data did not leave any trace on possible patient revelation. Further, the entire data extraction process was under constant monitoring and satisfaction of the ZCH's administration personnel.

### Data Collection and Analysis

Data was retrieved from the ART Clinic from April 2015 to May 2015 by trained data clerks at the hospital and was checked and cleaned by trained personnel at the end of each month. After data was obtained, it was checked for completeness and clarity. A particular patient's record with missing key variables was excluded. The data was exported to excel for cleaning. However, statistical data analysis, coding and labelling of variables were done in SPSS software version 20 IBM for Windows. The entire data analysis process including running of our proposed statistical models were done in STATA software version 12.

Descriptive statistical analysis was done on patients' socio-demographic characteristics and ART regimens. These included all variables which were captured in the ART master cards at the time of regimen side effect appearance, namely age at which the side effect was first recorded; patient's place of residence; occupation; weight and height at the start of the therapy; and gender.

Secondly, tested by Cramer's V for its strength, a Chisquare analysis test (McHugh, 2012) was used to measure the levels of association between regimen effect types (peripheral neuropathy, lipodystrophy, cough, leg pains/numbness, lactic acidosis and severe skin rash and other symptoms) and the aforementioned explanatory variables.

Lastly, in order to measure the significance of explanatory variables on the identified regimen effect types, herein categorised as “multinomial response variable”, a multinomial logistic regression model (Schwab, 2002)


$$log(\frac{\pi_{ij}}{\pi_{ij^{*}}})=x_{i}^{T}\beta_{j}$$

was proposed where j* means the index for the baseline category for which *j* ≠ j* and *x*_*i*_'s are the explanatory variables included in the model together with their corresponding regression coefficients *β*_*j*_. The model formulation was supplemented with marginal effect analysis of the regression coefficients (Vittinghoff, Shiboski, Glidden, & McCulloch, 2004). All the analyses were considered at a 5% significance level.

## Results and Discussion

Out of the 901 patients who showed side effects, 65.33 percent were females. The mean age of the patients was 42 years with most of the population falling above the youth age band of 10 to 35 years. The average age of the males was higher than that of the females. Youths contributed to 30.33 percent of the patients, and the rest were over 35 years. For the youths, 77.29 percent were females while 60.13% of those over 35 years were females. Most patients were from Chikowi (45.67%) followed by Malemia (21.67%) and Kuntumanji, Mlumbe, and Mwambo were 13.56 percent, 9.89 percent and 9.22 percent, respectively.

Farmers constituted 30.22 percent of the patients and about 28.78 percent were those who run some kind of business. Students constituted four percent of the population and, further, housewives contributed 16 percent of the patients. At least half of the patients weighed below 50 Kgs. The majority (50.67%) of the patients had received 1A regimen, and 30.78 percent had received 2A regimen, and the rest (18.56%) had received 5A regimen. In terms of type of side effect, a patient ever had, only 6.78% had lipodystrophy while the rest had proportions of at least 10%. Those with other unspecified symptoms had the largest proportion (43.89%).

Although 1A regimen exhibited proportionally higher records of side effects than others, across the list of all the side effects, the contributed from lipodystrophy (5.04%) was less compared to lipodystrophy's contributions of 10.47 and 5.39 percent in 2A and 5A regimens, respectively. On the other hand, notable side effect of 2A regimen was less contributed by leg pains (9.75) as compared to 1A and 5A regimens which had 10.75 and 16.17 percent leg pain contributions, respectively as illustrated in Table 1.

**Table 1:** Distribution of ARV regimens by Side effect

ARV Regimen	Side Effect						

	Lipodystrophy N (%)	Peripheral neuropathy N (%)	Skin rash N (%)	Other Symptoms N (%)	Leg pains N (%)	Cough N (%)	Total N (%)
1A	23 (5.04)	66 (14.47)	63 (13.82)	209 (45.83)	49 (10.75)	46 (10.09)	456
2A	29(10.47)	42 (15.16)	29 (10.73)	117 (42.24)	27 (9.75)	33 (11.91)	277
5A	9(5.39)	25 (14.97)	23 (13.77)	69 (41.32)	27 (16.17)	14 (8.38)	167
					103		
**Total**	61 (6.77)	133 (14.78)	115 (12.78)	395 (43.89)	(11.44)	93 (10.33)	900

Through descriptive analysis, some variation could be noted between the socio-demographic characteristics and the side effects (see Table 2). For instance, 14.42% of males against 9.86% of females had experienced legs pains. Similarly, 18.59% of male patients had experienced peripheral neuropathy as compared to 12.76% females. The trend, however, was not always in favour of females as some side effects could be observed in higher proportions for females than males. For example, unlike men, women are more likely to have side effects like cough, peripheral neuropathy and leg pains compared to lipodystrophy with old (50+ years) people being less likely to get skin rash and other symptoms compared to lipodystrophy. It would be of interest then to examine the influence of physiological structures of males and females on regimen side effects (Horstmanshoff, King, & Zittel, 2012). Regimen side effects explained at endoscopy level should be more pronounced.

**Table 2:** Socio-demographic characteristics of respondents by Side Effect

		Side Effect					
		
Characteristics	Total (N)	Lipodystrophy	Peripheral neuropathy	Skin rash	Leg pains	Cough	Other symptoms
**Total**	**900**	**61**	**133**	**115**	**103**	**93**	**395**
**Sex**							
Female	588	7.99	12.76	13.95	9.86	10.2	45.24
Male	312	4.49	18.59	10.58	14.42	10.58	41.35
**Age Group**							
14-30	121	5.79	8.26	18.18	8.26	14.05	45.45
31-40	324	5.25	11.42	13.89	10.49	10.19	48.77
41-50	266	6.77	17.67	12.03	10.53	10.15	42.86
51-80	189	10.05	20.63	8.47	16.4	8.47	35.98
**Weight**							
25-50	463	6.7	14.04	15.12	11.88	9.5	42.76
51-60	295	7.46	15.59	12.2	9.49	11.53	43.73
61+	142	5.63	15.49	6.34	14.08	10.56	47.89
**Height**							
143-150	195	5.64	11.79	14.87	13.85	12.31	41.54
151-160	458	7.21	15.72	12.88	11.57	8.95	43.67
161+	247	6.88	15.38	10.93	9.31	11.34	46.15
**Occupation**							
Business	259	7.34	15.44	12.74	13.51	10.04	40.93
Farmer	272	4.04		11.76	12.13	10.29	49.63
House wife	144	7.64	20.14	8.33	10.42	11.81	41.67
Other	193	8.81	13.47	16.06	8.29	9.84	43.52
Student	32	9.38	15.63	21.88	12.5	9.38	31.25
**Youths**							
Youth (10-35)	273	6.59		15.02	10.26	13.55	46.89
Other (36+)	627	6.86	17.86	11.8	11.96	8.93	42.58

**Figure 2:**
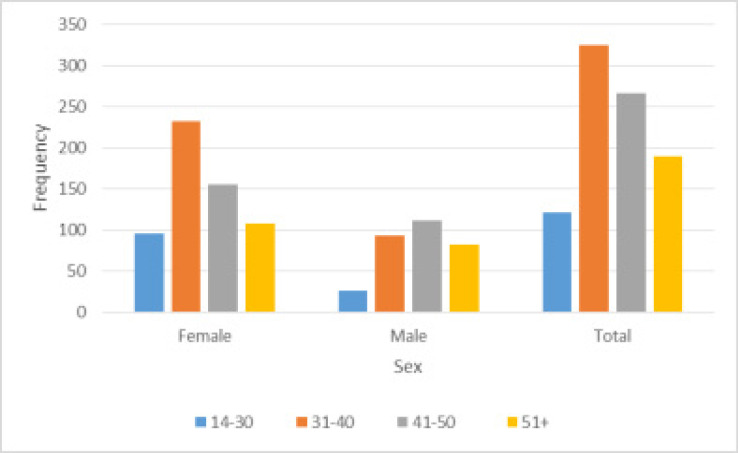
ARV regimen side effects age distribution by sex

Further, considerable large differences were also observed for variables age and residence. Of the 273 youths, 7.69 percent ever had peripheral neuropathy as compared to 17.86 percent, about 10 percentage points higher, of individuals aged 36 years and over. Perhaps, these findings provide a signal that when formulating policies and decisions regarding use of and switching of ARV drugs, not only people of old age should be at forefront of consideration, but minors also. But, with respect to sex and age, Figure 2 shows that the majority (39.39%) of the women that showed side effects were in the age range of 31 to 40. The least percentage was 16.13, contributed by those that were in the age range of 14 to 30. Comparably, 35.58 percent of men showed side effects with a majority coming from the age range of 41 to 50 and the least (8.33%) from the age group 14 to 30.

**Figure 3:**
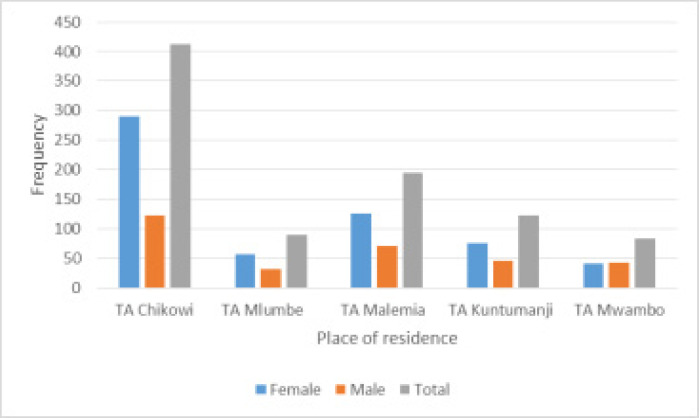
ARV regimen side effects distribution by residence by gender

Going by gender, we found that majority of men and women, ever to have experienced regimen side effects, came from T/A Chikowi, 39.10% and 49.24% respectively (Figure 3). However, we do not attribute the regimen effects experiences to place of residence due to limited knowledge of the socio-and-economic differences of the five T/As in Zomba. We did not find any health economic data to have ever been compiled specifically on the population of ZCH's catchment area. Limited by such data, we suggest that other studies be done to clearly establish economic differences of areas where patients on ART come from, not only in Zomba, to be able to make any proper statement about relating regimen symptoms or side effects with location.

That said, findings of Figures 2 and 3 were found to be in line with the Chi-square test of association results (Table 3) in which sex ($X_{\;5}^{2}=14.64,p<0.05$), age group ($X_{15}^{2}=34.50,p<0.05$) and age structure ($X_{\;5}^{2}=20.08,p<0.01$) of study participants are also strongly associated with ARV regimen symptoms. Our results are therefore in excellent agreement with Subbaraman et al (2007) who that showed that ARV regimen side effects varied across areas. Similarly, a previous study by Palmisano and Vella (2011) indicated that patients' occupation and income levels are also associated with the side effects.

**Table 3:** Results of Chi-square analysis between characteristics of respondents and side effects

			Side Effects								
			
		Total (N)	Lipodystrophy	Peripheral neuropathy	Skin rash	Leg pains	Cough	Other symptoms	Chi-Square	*df*	*p value*
		900									
**Percentage**	**Sex**								14.636	5	0.012*
65.33	female	588	7.99	12.76	13.95	9.86	10.2	45.24			
34.67	male	312	4.49	18.59	10.58	14.42	10.58	41.35			
	**Age Group**								34.5037	15	0.003*
13.44	14-30	121	5.79	8.26	18.18	8.26	14.05	45.45			
36.00	31-40	324	5.25	11.42	13.89	10.49	10.19	48.77			
29.56	41-50	266	6.77	17.67	12.03	10.53	10.15	42.86			
21.00	51-80	189	10.05	20.63	8.47	16.4	8.47	35.98			
	**Weight**								10.8019	10	0.373
51.44	25-50	463	6.7	14.04	15.12	11.88	9.5	42.76			
32.78	51-60	295	7.46	15.59	12.2	9.49	11.53	43.73			
15.78	61+	142	5.63	15.49	6.34	14.08	10.56	47.89			
	**Height**								7.6745	10	0.661
21.67	143-150	195	5.64	11.79	14.87	13.85	12.31	41.54			
50.89	151-160	458	7.21	15.72	12.88	11.57	8.95	43.67			
27.44	161+	247	6.88	15.38	10.93	9.31	11.34	46.15			
	**Occupation**								22.5352	20	0.312
28.78	business	259	7.34	15.44	12.74	13.51	10.04	40.93			
30.22	farmer	272	4.04	12.13	11.76	12.13	10.29	49.63			
16.00	house wife	144	7.64	20.14	8.33	10.42	11.81	41.67			
21.44	other	193	8.81	13.47	16.06	8.29	9.84	43.52			
3.56	student	32	9.38	15.63	21.88	12.5	9.38	31.25			
	**Age structure**								20.0888	5	0.001*
30.33	Youth (10-35)	273	6.59	7.69	15.02	10.26	13.55	46.89			
69.67	Other (36+)	627	6.86	17.86	11.8	11.96	8.93	42.58			

**Figure 4:**
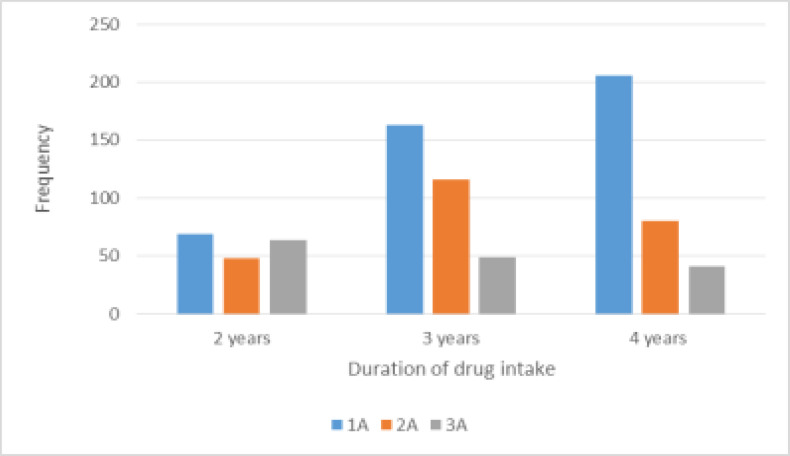
Distribution of ARV regimen side effects by regimen types by duration of intake

Further, results of Figure 4 suggest that patients who had stayed on therapy for 3 to 4 years were ore likely to show side effects than those who had been on therapy for 2 or less years. This might explain why at such an extent of our study, still 1A regimen had more records of pronounced ARV side effects than 2A and 5A. Although it rendered all other variables insignificant when included in the multinomial logistic regression model, duration of drug intake (p<0.01) showed significant association with ARV regimen symptoms. Thus, a more profound study would be to compare the effects of the three regimens for patients over an equal duration of drug intake.

Additionally, Figure 4 shows that with an increase in duration of drug intake (2 to 4 years), the distribution of side effects for 2A regimen is bell-shaped, almost normal. However, while the side effects are escalating for 1A regimen, a slight decrease in effects is observed for 5A regimen. The results could explain the possible reasons why patients at the studied ART clinic were switched to 2A and 5A regimens. The results therefore provide a highlight to our first research question which sought to quantify if there exist significance differences between type 1A regimen and types2A and 5 A regimens. Clearly, Figure 4 shows that type 1A regimen side effects prolong with duration of drug intake, thereby posing serious challenges to health of the patients. However, the behaviour of 2A regimen side effects with duration of intake attracts an attention of a pharmaceutical study on each drug to establish an optimal duration of intake for each type of regimen. The pharmaceutical findings will be very useful to health policy makers and drug manufacturers.

Although we could not find any significant association between proportion of regimen symptoms and people's occupation, farmers reported more on other symptoms (49.63%) as compared to people of other occupations. This is in line with earlier studies which found that farmers tend not to adhere to ART procedures because they are often occupied (Gast & Mathes, 2019). This result may somehow agree with a study by Torres-Madriz et al. (2011) who showed that presence of workplace accommodations is significantly associated with adherence (p <0.05). This may probably mean the reported ARV regimen symptoms are indirectly related to the human behaviour also such as cognitive behaviour (Corless et al., 2005; Weber et al., 2004).

Results of the combined effect of age, sex and weight (explanatory variables) on the ARV regimen side effects (response variable) in the multinomial logistic regression model (Table 4) show that, except for cough (p-value = 0.046, CI= [1.0148-4.4969]), peripheral neuropathy (p=0.010, CI= [1.2447-5.0154]), and leg pains/numbness (p=0.008, CI= [1.2993-5.5046]), gender does not significantly contribute to the observed differences in symptoms appearance. Particularly, on the appearances of the lipodystrophy (p-value = 0.073) and skin rash to lipodystrophy (p = 0.229) symptoms. “Lipodystrophy” was considered as the base outcome or reference outcome for the model. Nonetheless, the outcome that gender is significant in explaining the much-observed differences in the ARV regimen symptoms appearances is in agreement with study findings of Palmisano and Vella (2011). In addition, our study is also broadly consistent with literature survey of Coleman et al. (2009) that the prevalence of lipodystrophy substantially correlates with age (p< 0.05) due to either ARV medicines the patients take (Caron-Debarle, Lagathu, Boccara, Vigouroux, & Capeau, 2010) or the disease itself (WebMD, 2021). However, in children (<18 years), Gupta et al. (2017) found that lipodystrophy condition may be non-HIV-related. We do not rule out such possible cases in our study as well.

**Table 4:** Multinomial logistic regression coefficients of side effects by the explanatory variable

Side Effect	RRR	*p-value*	95% Confidence Interval
**Cough**			
male	2.136	0.046	(1.0148, 4.4969)
age	0.959	0.006	(0.9297, 0.9882)
wgt	1.014	0.462	(0.9766, 1.0535)
cons	3.381	0.292	(0.3512, 32.5474)
**Peripheral Neuropathy**			
male	2.499	0.010	(1.2447, 5.0154)
age	1.009	0.540	(0.9813, 1.0368)
wgt	1.011	0.570	(0.9747, 1.0476)
cons	0.640	0.686	(0.0739, 5.5487)
**Severe Skin Rash**				
male	1.567	0.229	(0.7538, 3.2587)
age	0.962	0.009	(0.9344, 0.9905)
wgt	0.980	0.310	(0.9429, 1.0188)
cons	22.992	0.006	(2.4435, 216.3337)
**Leg Pains (Numbness)**			
male	2.674	0.008	(1.2993, 5.5046)
age	0.992	0.569	(0.9633, 1.0208)
wgt	1.015	0.433	(0.9779, 1.0535)
cons	0.814	0.857	(0.0869, 7.6291)
**Other Symptoms**			
male	1.797	0.073	(0.946, 3.4151)
age	0.973	0.029	(0.9488, 0.9971)
wgt	1.010	0.544	(0.9779, 1.0433)
cons	10.553	0.016	(1.5458, 72.0423)

*Lipodystrophy was considered as the base outcome in the model

In general, the risk ratio estimates suggest that males unlike females are more likely to get a cough as compared to lipodystrophy. Similarly, females unlike males are more likely to have peripheral neuropathy compared to lipodystrophy. The chances of getting leg pains to lipodystrophy is higher in females as compared to males (RRR=2.674). Such an observation of gender disparities associated with different risk levels is also documented in a study previously done by (NIH, 2020).

Further, holding all other variables constant, age significantly explained the differences between other symptoms and lipodystrophy (p = 0.029, CI= [0.9488- 0.9971]). Older people have less chances of getting other symptoms compared to lipodystrophy than people in the younger ages (RRR=0.973). The results also suggests that older people are less likely to get a cough and skin rash (RRR=0.959) compared to lipodystrophy than those in the younger ages. However, age does not significantly explain the differences between leg pains and lipodystrophy (p =0.506) as well as peripheral neuropathy and lipodystrophy (p =0.928). However, weight did not show any significant role in explaining the difference between lipodystrophy and any of the side effects.

It is not uncommon to mistakenly interpret the coefficient as meaning that the probability of, say, “coughing” for males is higher. An appropriate approach to come around this is to calculate marginal effects (Vittinghoff et al., 2004). Average marginal effect of male on Lipodystrophy was found to be-0.0394 (Table 5). This means that the probability of having Lipodystrophy, as a side effect, is on average about four percentage points lower for males than for females with the same age and weight.

**Table 5:** Marginal effects of coefficients of the multinomial model

Pr (Side Effect)	Variable With Respect to:		

	Age	Weight	Male
Other Symptoms	-0.0028 (0.068)	0.0015 (0.415)	-0.0273 (0.437)
Cough	-0.0021 (0.027)	0.0008 (0.475)	0.0118 (0.595)
Peritheral			
Neuropathy	0.0042 (0.000)	0.0005 (0.692)	0.0396 (0.118)
Lipodystrophy	0.0014 (0.061)	-0.0004 (0.661)	-0.0394 (0.015)
Skin Rash	-0.0021 (0.041)	-0.0033	-0.0241 (0.298)
Leg pains	0.0014 (0.141)	0.0009 (0.432)	0.0394 (0.092)

Further, it was observed that the probability of either having cough (-0.0021, p value =0.027), or skin rash (-0.0021, p =0.041), as a side effect, on average, decreased as age increased with the same sex and weight. The probability of having peripheral neuropathy (0.0042, p< 0.001), however, increases with age. Such may be the case because the incidence of peripheral neuropathy is widely reported to increase with age (Anish, Nagappa, Mahadevan, & Taly, 2015) such that the disorder sometimes interferes with daily activities leading to increased risk of falls and injury among people of old age (Goss et al., 2011). Thus, our results are in perfect agreement with findings of Mao et al. (2019) and Popescu et al. (2016). Age is an independent risk factor for the development of diabetic peripheral neuropathy.

Although weight is statistically insignificant, the risk of having skin rash (-0.0033, p =0.019) decreases as weight increases for the same age and sex. This result may sound contrary to what Mori et al. (2017) found in their study; that among females, obesity is highly correlated with skin disorders such as increase in surface roughness; reduction in water content; reduction of [NP]-type ceramide; and changes in the lipid profile of stratum corneumceramide. Moreover, substantial redness accompanied by a 34% increase in skin blood flow was observed in obese women. Nonetheless, according to Enwereji et al. (2020), gaining of body weight due to good nutrition guarantees excellent health in HIV infection and minimizes risks to various symptoms. The observation by Enwereji et al. (2020) leads us to conclude that our finding of inversely relating probability of developing skin rush and body weight has to do with nutritional address on the regimen effects. It is therefore recommended that patients taking different ARV regimens be put under good diet to gain weight while seriously observing and controlling their obesity.

## Limitations

This study had some limitations related to the design and data collection. The study's data was collected from one hospital which precluded a more definitive conclusion. In order to properly uncover the issues addressed here more broadly, it would therefore be proper that another study be done on the same topic or with a similar angle but involving a large sample from multiple data sources housing ART records. While we tried our best to recruit the much representative sample, our sample size was also affected by the decision of dropping all patients' IDs with missing key variables. There is also a potential for recall bias since some information about patients was given by their guardians.

The study did not include economic status and wellbeing (diet) of study participants as variables that might have contributed to the side effects. A report by (Avert, 2012) showed that good diets can lessen ARV regimen side effects. Probably, if diets were included in testing the factors that affect ARV side effects, the results would be more accurate. In future data which is captured in hospitals and surveys should therefore include questions that capture information on people's diets and eating habits.

## Conclusion and Recommendations

ARVs have adverse side effects (Carr & Cooper, 2000), such that earlier on, the effects were among the most common reasons for switching or discontinuing therapy and for medication non-adherence. Studies conducted by Subbaraman et al (2007) and Palmisano and Vella (2011) established that ARV regimens were the major cause of most of the bone and heart complications in HIV patients and that the risks varied basing on the drug combinations (regimens). Besides the individual's comorbidities, concomitant medications, and prior history of drug intolerances, knowledge of potential adverse effects, is of great importance for the clinicians when selecting an ARV regimen (Max & Sherer, 2000). However, such evidence-based studies in Malawi were lacking. In this article, we have therefore evaluated the side effects of 1A, 2A and 5A as well as investigated on other factors that may contribute to side effects of patients who were on ARV treatment through a retrospective study that was based on the review of four-year (2011-2015) hospital records of Zomba Central Hospital.

We found that 1A regimen had the highest frequencies in almost all the side effects as compared to 2A and 5Awhich only contributed 30.85% and 18.53%, respectively. However, analysis showed that ARV regimens were not really significant in explaining side effects. The side effects were explained by other factors other than ARVs, contradicting with the claim that was made by the Ministry of Health (2013) that patients on 1A had the greatest risks of having side effects compared to 2A and 5A. Further, the study showed that age (p-value = 0.003), gender (p-value = 0.012) and occupation (p-value = 0.001) were significantly associated the reported regimen symptoms. It was shown that women unlike men are more likely to have side effects like cough, peripheral neuropathy and leg pains compared to lipodystrophy with old (50years+) people being less likely to get skin rash and other symptoms compared to lipodystrophy. On top of that, the descriptive analytical findings showed high frequencies of women in most side effects as compared to men. For example, the chances of getting leg pains to lipodystrophy is higher in females as compared to males (RRR=2.674). These study findings therefore help in identifying high-risk sub-populations and areas to be targeted for PEP, treatment and educative campaigns (Emina et al., 2013).

Therefore, we recommend that when recruiting HIV patients for different ARV regimens in Malawi, extra attention should be given to women as they are more likely to get side effects than their counterparts (see Figure 2). Besides, based on the 2015–2016 Malawi Demographic and Health Survey (MDHS) and AIDS Indicator Survey data, a recent study by Nutor et al. (2020) has shown that HIV prevalence rate is high in women (10.8%) than men (7.0%) in Malawi. To the contrary, much attention is given to young people (less than 14 years) in most hospitals with an assumption that most adults are responsible. However, women are believed to be care takers and yet the majority of them in the country are reported as either without proper working qualification or never to have attended school (National Statistical Office, 2015). It is therefore important that women know all the risks associated with ARVs, and actions that can help lessen them. They also need to know the foods that help in lessening side effects as it is mostly women who decide what to eat in homes (Beardsworth et al., 2002; Shiva, 2009). With this, not only will the side effects be lessened in women but also in men.

Lastly, peoples' health affects national development (Musgrove, 1993). As noted in this study, most of the people that have side effects are in the productive age group ([31-40]; 39.39%, see Figure 1) thus affecting national development greatly (Mwale, 2002). It is important, therefore, that much focus should be put in reducing the rate at which HIV is spreading as ARVs do not guarantee a 100% side effect free life.
